# A web-based dynamic nomogram for rupture risk of posterior communicating artery aneurysms utilizing clinical, morphological, and hemodynamic characteristics

**DOI:** 10.3389/fneur.2022.985573

**Published:** 2022-09-14

**Authors:** Heng Wei, Wenrui Han, Qi Tian, Kun Yao, Peibang He, Jianfeng Wang, Yujia Guo, Qianxue Chen, Mingchang Li

**Affiliations:** ^1^Department of Neurosurgery, Renmin Hospital of Wuhan University, Wuhan, China; ^2^Department of Neurosurgery, Jingzhou Central Hospital, Jingzhou, China

**Keywords:** dynamic nomogram, posterior communicating artery aneurysms, rupture risk, LASSO regression, external validation

## Abstract

**Background:**

Predicting rupture risk is important for aneurysm management. This research aimed to develop and validate a nomogram model to forecast the rupture risk of posterior communicating artery (PcomA) aneurysms.

**Methods:**

Clinical, morphological, and hemodynamic parameters of 107 unruptured PcomA aneurysms and 225 ruptured PcomA aneurysms were retrospectively analyzed. The least absolute shrinkage and selection operator (LASSO) analysis was applied to identify the optimal rupture risk factors, and a web-based dynamic nomogram was developed accordingly. The nomogram model was internally validated and externally validated independently. The receiver operating characteristic (ROC) curve was used to assess the discrimination of nomogram, and simultaneously the Hosmer–Lemeshow test and calibration plots were used to assess the calibration. Decision curve analysis (DCA) and clinical impact curve (CIC) were used to evaluate the clinical utility of nomogram additionally.

**Results:**

Four optimal rupture predictors of PcomA aneurysms were selected by LASSO and identified by multivariate logistic analysis, including hypertension, aspect ratio (AR), oscillatory shear index (OSI), and wall shear stress (WSS). A web-based dynamic nomogram was then developed. The area under the curve (AUC) in the training and external validation cohorts was 0.872 and 0.867, respectively. The Hosmer–Lemeshow *p* > 0.05 and calibration curves showed an appropriate fit. The results of DCA and CIC indicated that the net benefit rate of the nomogram model is higher than other models.

**Conclusion:**

Hypertension, high AR, high OSI, and low WSS were the most relevant risk factors for rupture of PcomA aneurysms. A web-based dynamic nomogram thus established demonstrated adequate discrimination and calibration after internal and external validation. We hope that this tool will provide guidance for the management of PcomA aneurysms.

## Introduction

Due to the broader availability of non-invasive imaging techniques, the detection rate of unruptured intracranial aneurysms (UIAs) is getting higher (1). Since endovascular or surgical treatments of UIAs may cause complications during the procedure (2), the treatment strategy for UIAs remains a controversial topic. PcomA aneurysm not only has a high incidence but also has a high treatment challenge due to the PcomA special structure. Therefore, the assessment of the rupture risk of PcomA aneurysms is essential to guide treatment.

Assessing the rupture risk of aneurysms has always been a research hotspot, but most of the previous assessments are based on traditional statistical methods. Although such models are simple and robust, they are limited to relatively few features and assume that there is a linear relationship between each feature and rupture risk. Machine learning can make up for this defect. Machine learning is a group of algorithms that function to train a computer to learn complex non-linear relationships by observing a large amount of data. It is the core of artificial intelligence. Machine-learning models allow for a more flexible relationship between features and risks and have more complex characteristics than the traditional statistical models. Classical machine learning algorithms, such as support vector machine, random forest, and decision tree, can use multiple characteristics of aneurysms as input variables to build an excellent prediction model. Ou et al. found that the prediction effect of the extreme gradient boosting model is the best after comparing a variety of machine learning models. Also, the model is well explained by shapley additive explanations analysis, which overcomes the “black boxes” problem of machine learning (3). Artificial neural networks can extract more inherent features than classical machine learning. Previous studies have shown that the use of a convolutional neural network depth learning algorithm, by extracting 3D-DSA multi-view image information to predict the rupture risk of aneurysms <7 mm, has high sensitivity and accuracy (4). The accuracy of machine learning is based on the collection of a large amount of data, but it is sometimes difficult to collect a large amount of unprocessed aneurysm information. Through the self-supervised learning framework, many unlabeled data are pre-trained, and in the case of insufficient data, a model with good prediction results can also be established (5).

Previous studies have reported that the clinical, morphological, and hemodynamic parameters of patients with aneurysms can predict the rupture risk of IAs (6, 7). However, the analysis revealed the low discrimination and calibration of the simple model, which may be insufficient for clinical risk assessment (8). In contrast to other models, a nomogram does not require the conversion of a continuous variable to a categorical variable, while the associated probabilities of multiple variables can be unified in a single nomogram. For complex models, such as survival models that include time predictors, nomograms can also be used to demonstrate (9). This study aimed to develop and validate a nomogram for rupture risk assessment of PcomA aneurysms based on clinical, morphological, and hemodynamic features.

## Materials and methods

### Patient population enrollment

We retrospectively reviewed the medical records and cerebrovascular images of 332 consecutive patients with PcomA aneurysms admitted to Renmin Hospital of Wuhan University from January 2015 to December 2021, and the data was used to develop the nomogram model. In addition, 96 consecutive patients with PcomA aneurysms were admitted to another independent hospital from January 2018 to December 2021 for external validation of the nomogram model. This retrospective study was approved by the clinical research ethics committee of the two hospitals.

Inclusion criteria: (1) saccular aneurysm of the posterior communicating artery; (2) complete clinical medical records. Exclusion criteria: (1) patients with malignant tumors, severe systemic disease; (2) pseudoaneurysm, inflammatory aneurysms, traumatic aneurysms, dissecting aneurysms, and fusiform aneurysms; (3) aneurysms combined with MoyaMoya disease, vascular malformations, arteriovenous fistulas; (4) multiple aneurysms; (5) images with poor quality for morphology measurement or computational fluid dynamics.

### Patient groups

Patients were divided into the ruptured and unruptured groups based on subarachnoid hemorrhage (SAH). SAH was confirmed by computed tomography (CT) of the brain and clinical features. Lumbar puncture was considered mandatory to confirm the diagnosis in cases where SAH was suspected clinically, but brain CT was negative (10). Two neurosurgeons with over 10 years of experience in interpreting cerebral angiograms and the endovascular treatment of IAs confirmed the findings.

### Imaging

Transfemoral artery catheterization was used for all catheter angiographies. 3D images were obtained through DSA using a Siemens angiographic system (Siemens Healthineers, Forchheim, Germany). In contrast, rotational angiograms were performed 2 s after 5-s contrast injection with 18 ml of contrast agent at the rate of 3 ml/s and a 200° rotation. The images were reconstructed on the workstation into a 3D model and then exported in digital imaging and communications in medicine format.

### Aneurysm modeling

Image data were imported into the software Mimics medical 21.0 (Materialize, Leuven, Belgium) to segment geometries and construct the preliminary 3D models and then imported into 3-matic medical 13.0 (Materialize, Leuven, Belgium) in STL format for model repair and smoothing. The software ANSYS Fluent 2021 R2 and CFD-post 2021 R2 (ANSYS Inc., USA) were used to simulate hemodynamics and calculate the value of each hemodynamic parameter. Model mesh division was carried out in Fluent, controlling the maximum size of poly-hexcore mesh at 0.1 mm. Six boundary layers were specially set during mesh generation to accurately obtain the hemodynamic parameters of the model. The thickness of the first layer was 0.01 mm and layer growth rate was set to 1.2. Each geometry was meshed to generate 0.5 million−1.5 million volume elements for subsequent fluid dynamics computation. The pulsating velocity waveform measured by transcranial Doppler ultrasound was used as the inflow boundary condition. The flow waveform was scaled to obtain the mean internal carotid artery (ICA) inlet flow rate of 4.6 ml/s under pulsating conditions. The outlet was set at zero pressure and the flow rate through each outlet artery was proportional to the cube of its diameter. The vessel was modeled as a rigid wall, and the blood flow was modeled as an incompressible Newtonian fluid with constant temperature and laminar flow. A density of 1,060 kg/m^3^ and a dynamic viscosity of 0.0035 Pa·s were specified for each simulation. Monitor convergence absolute criterion was set to ≤1 × 10^−4^. Finally, the calculation time was set to last for three cardiac cycles, with the result of the third cycle reaching a stable state after 200-time steps. Three pulsatile cycles were simulated to ensure that numerical stability had been achieved, and the last cycle was taken as the output. All data presented were time averages over the third pulsatile cycle of the flow simulation.

### Clinical characteristics

The following clinical characteristics were collected from the medical records system of each patient: name, gender, age, smoking history, alcohol consumption, hypertension, diabetes, hyperlipidemia, and coronary heart disease (CHD).

### Morphology analysis

The 3D model of the aneurysm and parent vessels could be measured in our workplace system. We measured and calculated the following parameters of the aneurysm: the maximum diameter (D): the maximum distance from the center of the aneurysm neck to a point on the sac; the maximum width (W): the maximum distance between two points in the aneurysm sac perpendicular to the maximum diameter of the aneurysm; neck width (N): the maximum diameter in the neck plane; height (H): the maximum distance from the plane of the neck to the surface of the aneurysm; aspect ratio (AR = D/N): the ratio of the diameter of the aneurysm to the width of the aneurysm neck; irregular shape: aneurysms with an irregular shape defined as having lobular or daughter sacs; height-to-width ratio (H/W): the ratio of the height of the aneurysm to the width of the aneurysm; and bottleneck factor (BNF = W/N): the ratio of the width of the aneurysm to the width of the aneurysm neck. Morphological parameters were defined and calculated as described in previous studies (11–13). The parameters were determined by two neuroradiologists who were blinded to patient information and stability status.

### Hemodynamic analysis

Six commonly studied hemodynamic parameters defined by the aneurysm surface and volume were calculated, including the wall shear stress (WSS), normalized wall shear stress (NWSS), wall shear stress gradient (WSSG), low shear area (LSA), intra-aneurysmal pressure (IAP), and oscillatory shear index (OSI). WSS, NWSS, WSSG, LSA, IAP, and OSI were calculated as average values. WSS refers to the tangential, frictional stress caused by the action of blood flow on the vessel wall; NWSS represents the ratio of the aneurysm wall shear force to the mean value of the parent artery wall shear force; WSSG indicates the amplitude of variation along the direction of the wall shear force; LSA was defined as the area of aneurysm wall exposed to the WSS below 10% of the mean WSS of the parent artery; IAP is the force energy with which blood strikes the inner wall of the aneurysm sac; OSI is a non-dimensional parameter and defined as a function of the change in the direction of the shear force at a particular location in the cardiac cycle. All the hemodynamic parameters were computed using the methods of previous reports (14–17).

### Statistical analysis

All data were analyzed using IBM SPSS Statistics version 25.0 (SPSS, Inc., Chicago, Illinois, US) and R software (version 3.6.3, R Foundation for Statistical Computing, Vienna, Austria). Continuous variables that conformed to normal distribution were analyzed using an independent *t*-test and presented as means ± SD; abnormal distributions were analyzed using the Mann–Whitney U-test and presented as medians with interquartile ranges. Categorical variables were expressed as numbers and analyzed using χ^2^ tests. The differences were considered statistically significant if the two-tailed *p*-values were <0.05 (95% confidence interval, CI). LASSO analysis and multivariate logistic regression analysis were used to identify independent risk factors to establish the nomogram for the risk of rupture of PcomA aneurysms.

To evaluate the performance of the nomogram, we used internal validation *via* a bootstrap method with 1,000 re-samples and external validation in an independent external cohort. The performance of the nomogram was measured by discrimination and calibration. Discrimination refers to the model's ability to distinguish patients with different outcomes. ROC curve and AUC were performed to assess the discriminatory capabilities, while C-index ≥0.70 indicates adequate discrimination (18). To reduce the impact of false positives or false negatives on discrimination, we also adopted the DCA and CIC test methods to confirm the clinical utility of the nomogram model (19, 20). Calibration refers to the consistency between the actual outcomes and predicted outcomes. The Hosmer–Lemeshow test and calibration plots were applied to evaluate the goodness of fit and calibration. The fit of the model was assessed by the Hosmer–Lemeshow test, and *p* > 0.05 was considered to indicate an appropriate fit. The plotted points closer to the 45° line indicated a better calibration (21).

We used the “foreign, glmnet, ggplot2” package in R software to generate the LASSO results and the “rms” to establish the nomogram. The “pROC, rmda, survival, ggplot2,” and “Resource Selection” packages were applied to generate the ROC, AUC, DCA, CIC, and calibration curve. The “shinyapps.io” and “DynNom” packages were used to develop a web-based dynamic nomogram.

## Results

### Clinical, morphological, and hemodynamic characteristics in training and external validation cohort

This study included 332 patients in the training cohort and 96 patients in the external validation cohorts. The average age of the training cohort was 58.55 ± 9.49 years, including 107 unruptured and 225 ruptured PcomA aneurysms. The average age of the external validation cohort was 60.36 ± 8.28 years, including 21 unruptured and 75 ruptured PcomA aneurysms. The clinical, morphological, and hemodynamic characteristics of participants in the training and external validation cohort are shown in Table 1. There were no significant differences between the training and the external validation cohort.

**Table 1:** Clinical, morphological, and hemodynamic characteristics in training cohort and external validation cohort.

**Characteristic**	**Training cohort** ** (*n* = 332)**	**External validation cohort** ** (*n* = 96)**	***P*-value**
Age (years)	58.55 ± 9.49	60.36 ± 8.28	0.090
Gender (female)	253 (76%)	78 (81%)	0.335
Hypertension	135 (41%)	37 (39%)	0.725
Diabetes	21 (6%)	2 (2%)	0.127
hyperlipidemia	31 (9%)	8 (8%)	0.843
CHD	18 (5%)	6 (6%)	0.801
Smoking	43 (13%)	13 (14%)	0.865
Drinking	23 (7%)	10 (10%)	0.278
Irregular shape	110 (33%)	33 (34%)	0.807
D (mm)	5.59 (4.26, 7.16)	6.03 (4.70, 7.26)	0.257
W (mm)	3.82 (3.14, 5.02)	4.12 (3.18, 4.72)	0.699
N (mm)	3.10 (2.52, 3.66)	3.15 (2.53, 3.73)	0.756
H (mm)	4.80 (3.57, 6.03)	4.72 (3.88, 5.82)	0.791
AR	1.85 ± 0.48	1.90 ± 0.33	0.348
BNF	1.25 (1.07, 1.51)	1.25(1.10, 1.37)	0.361
H/W	1.17 (1.04, 1.34)	1.24(1.09, 1.35)	0.076
OSI	0.031 (0.021, 0.041)	0.033(0.020, 0.047)	0.177
LSA	0.12 (0.08, 0.14)	0.12(0.08,0.15)	0.363
WSS (Pa)	3.14 (2.22, 4.10)	2.93(2.16, 3.47)	0.119
NWSS	0.67(0.59,0.74)	0.68(0.62,0.75)	0.448
WSSG	443.68(286.09, 624.94)	414.16(275.87, 593.22)	0.362
IAP (Pa)	404.54(319.30, 527.61)	384.85(305.05, 486.79)	0.142
Ruptured	225(68%)	75(78%)	0.275

CHD, coronary heart disease; D, maximum diameter; W, maximum width; N, neck width; H, height; AR, aspect ratio; BNF, bottleneck factor; H/W, height-to-width ratio; OSI, oscillatory shear index; LSA, low shear area; WSS, wall shear stress; NWSS, normalized wall shear stress; WSSG, wall shear stress gradient; IAP, intra-aneurysmal pressure.

### Univariable analysis of clinical, morphological, and hemodynamic factors of ruptured and unruptured PcomA aneurysms in the training cohort

In the training cohort, the univariate analysis results of clinical, morphological, and hemodynamic characteristics are shown in Table 2. The average age of patients in the ruptured group was 58.21 ± 9.15 years, including 52 men and 173 women. The average age of patients in the unruptured group was 59.25 ± 10.18 years, including 27 men and 80 women. In clinical characteristics, only hypertension showed a significant difference (*p* = 0.006). In morphological characteristics, the D (*p* < 0.001), W (*p* = 0.039), H (*p* = 0.001), AR (*p* < 0.001), BNF (*p* < 0.001), H/W (*p* = 0.001), and irregular shape (*p* = 0.001) were significantly different between the ruptured and unruptured group. In hemodynamic characteristics, the WSS (*p* < 0.001) and OSI (*p* < 0.001) showed significant differences.

**Table 2:** The clinical, morphological, and hemodynamic characteristics between ruptured and unruptured groups in the training cohort.

**Characteristic**	**Ruptured** ** (*n* = 225)**	**Unruptured** ** (*n* = 107)**	***P*-value**
Age (years)	58.21 ± 9.15	59.25 ± 10.18	0.350
Gender (female)	173 (77%)	80 (75%)	0.681
Hypertension	103 (46%)	32 (30%)	0.006
Diabetes	13 (6%)	8 (7%)	0.630
Hyperlipidemia	19 (8%)	12 (11%)	0.425
CHD	13 (6%)	5 (5%)	0.799
Smoking	32 (14%)	11 (10%)	0.383
Drinking	12 (5%)	11 (10%)	0.109
Irregular shape	88 (39%)	22 (21%)	0.001
D (mm)	6.11 (4.81, 7.29)	4.72 (3.44, 5.92)	<0.001
W (mm)	3.92 (3.24, 5.08)	3.52 (2.89, 4.89)	0.039
N (mm)	3.09 (2.52, 3.59)	3.13 (2.53, 3.86)	0.498
H (mm)	5.03 (3.95, 6.13)	4.32 (3.16, 5.32)	0.001
AR	1.99 ± 0.44	1.55 ± 0.42	<0.001
BNF	1.29 (1.11, 1.55)	1.17 (1.02, 1.33)	<0.001
H/W	1.19 (1.06, 1.37)	1.12 (1.00, 1.30)	0.001
OSI	0.03 (0.02, 0.04)	0.02 (0.01, 0.03)	<0.001
LSA	0.12 (0.08, 0.14)	0.11 (0.08, 0.14)	0.248
WSS (Pa)	2.94 (1.98, 3.45)	4.03 (3.23, 4.75)	<0.001
NWSS	0.67 (0.62, 0.73)	0.67 (0.59, 0.77)	0.598
WSSG	458.46 (323.29, 626.76)	432.61(270.11, 621.13)	0.263
IAP (Pa)	419.33 (325.99, 529.17)	389.27 (310.53, 513.37)	0.246

CHD, coronary heart disease; D, maximum diameter; W, maximum width; N, neck width; H, height; AR, aspect ratio; BNF, bottleneck factor; H/W, height-to-width ratio; OSI, oscillatory shear index; LSA, low shear area; WSS, wall shear stress; NWSS, normalized wall shear stress; WSSG, wall shear stress gradient; IAP, intra-aneurysmal pressure.

### Variable selection

**Figure 1:**
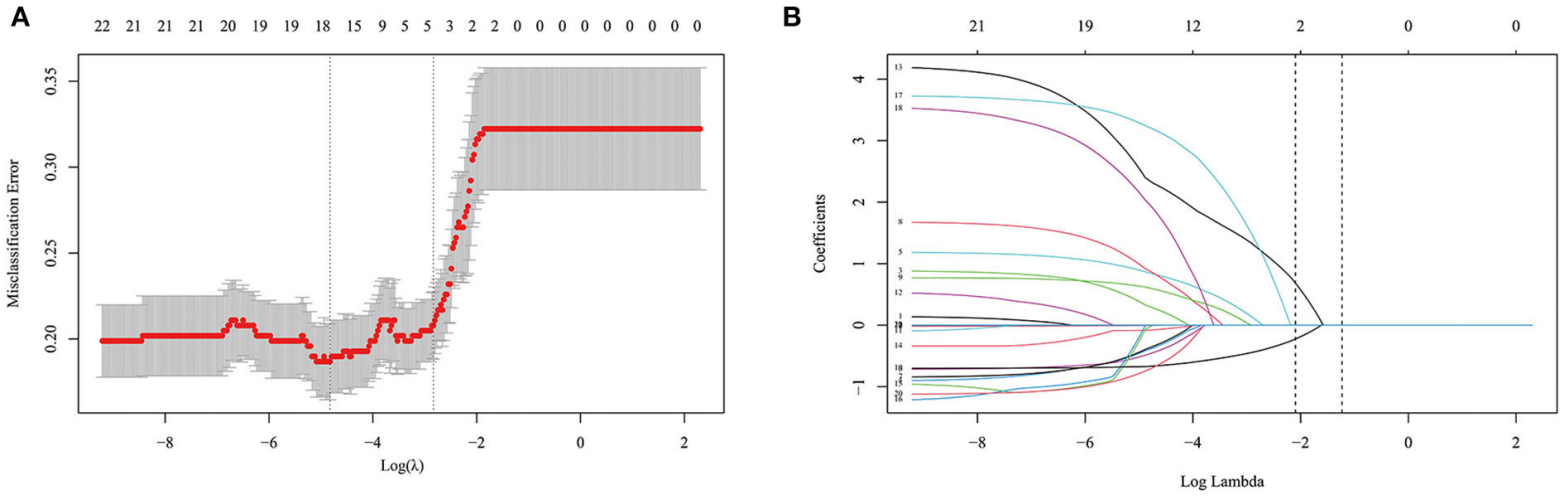
Selection of optimal variables by least absolute shrinkage and selection operator (LASSO) analysis. **(A)** The selection of optimal parameters (lambda) by 10-fold cross-validation. **(B)** The vertical line was plotted at the optimal λ of 0.058, with log (λ) = −2.834. Four factors with non-zero coefficients were finally selected.

Based on the R language, a LASSO model was constructed, and a 10-fold cross-test was performed. A total of 22 variables were shrunk to 17 when using the minimum error rate criterion. Finally, applying the one standard error (1-SE) criterion and adopting the optimal λ of 0.058 and log(λ) = −2.834, four factors with non-zero coefficients were finally selected by a 10-fold cross-validation to prevent overfitting (Figure 1). At the same time, the four parameters selected by LASSO analysis were applied in the multivariate logistic regression analysis, revealing that the four parameters, hypertension (OR = 2.631, 95% CI 1.400–4.944, *p* = 0.003), AR (OR = 9.937, 95% CI 4.726–20.892, *p* < 0.001), OSI (OR = 1.449, 95% CI 1.167–1.799, *p* = 0.001), and WSS (OR = 0.484, 95% CI 0.374–0.626, *p* < 0.001) were independent rupture risk factors (Table 3).

**Table 3:** Multivariable logistic regression analysis for the selected variables by LASSO.

**Variable**	**OR (95%, CI)**	***P*-value**
Hypertension	2.631 (1.400–4.944)	0.003
AR	9.937 (4.726–20.892)	<0.001
OSI ( ×100)	1.449 (1.167–1.799)	0.001
WSS	0.484 (0.374–0.626)	<0.001

AR, aspect ratio; OSI, oscillatory shear index; WSS, wall shear stress.

### Nomogram models development

**Figure 2:**
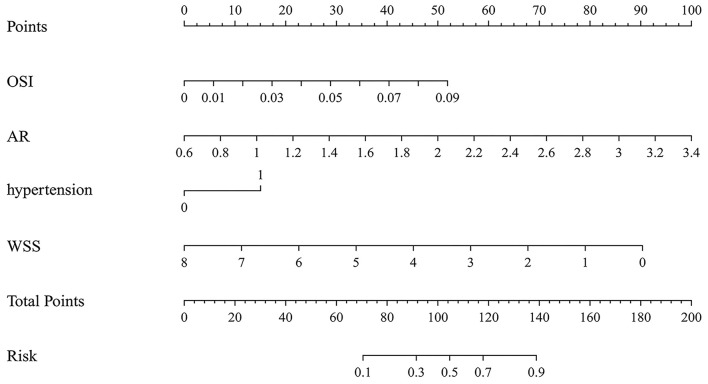
The nomogram model predicts the rupture risk of PcomA aneurysms, based on OSI, AR, hypertension, and WSS. OSI, oscillatory shear index; AR, aspect ratio; WSS, wall shear stress.

The variables identified by LASSO analysis were used to construct the nomogram model for predicting the rupture risk of PcomA aneurysms (Figure 2). Each of the risk factors in the nomogram was projected upward to a point. Hypertension had two classified variables, which were divided into 0 and 1 (1 = hypertension). AR had continuous values ranging from 0.6 to 3.4. OSI had continuous values ranging from 0 to 0.09. WSS had continuous values ranging from 0 to 8. Each of these was assigned points. The total score obtained by adding the respective scores of these four variables was converted into the rupture risk of PcomA aneurysms. A higher total score indicates a higher rupture risk of a PcomA aneurysm.

### Internal and external validation of a nomogram

**Figure 3:**
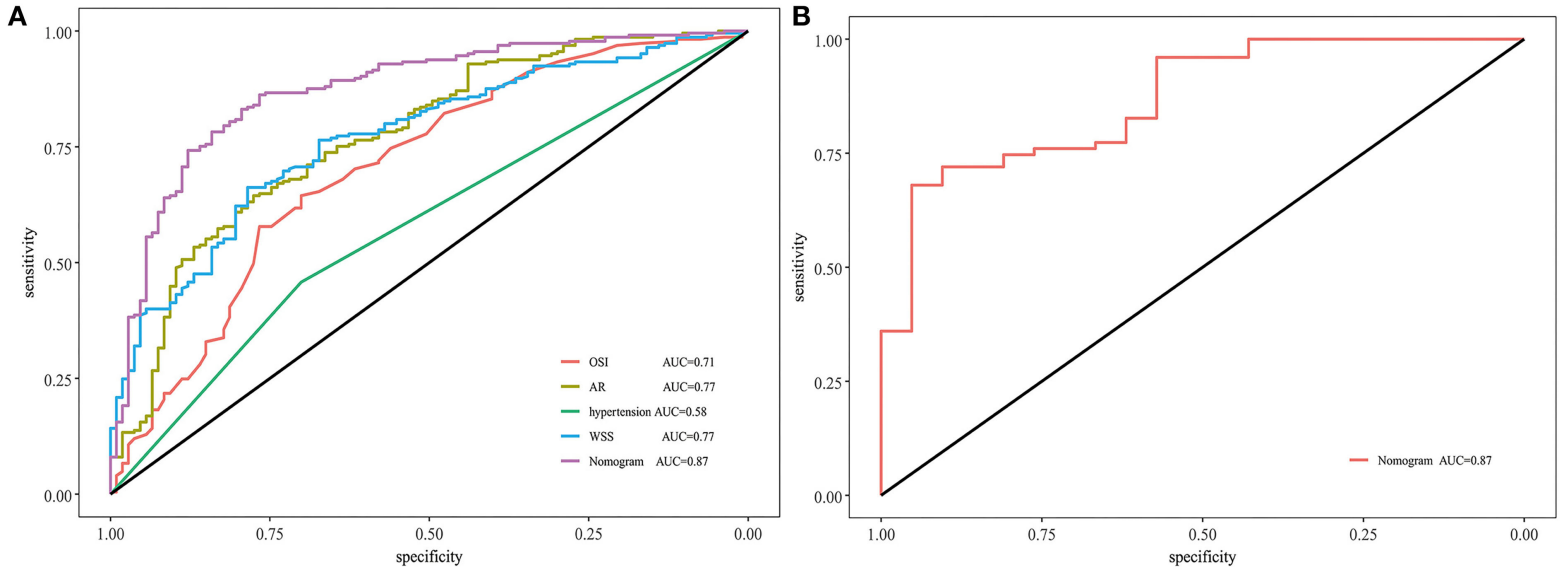
ROC and AUC analysis for nomogram validation. **(A)** Internal validation. **(B)** External validation. ROC, receiver operating characteristic; AUC, area under the curve; AR, aspect ratio; OSI, oscillatory shear index; WSS, wall shear stress.

**Figure 4:**
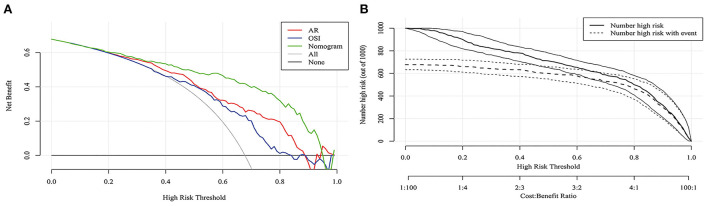
DCA and CIC curves of nomogram in the training cohort. **(A)** DCA curve. **(B)** CIC curve. DCA, decision curve analysis; CIC, clinical impact curve.

**Figure 5:**
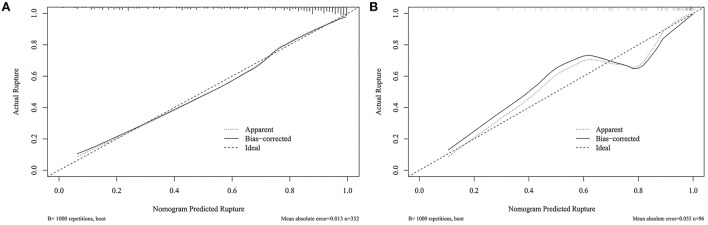
Calibration curve for nomogram validation. **(A)** Internal validation. **(B)** External validation.

Figure 3A shows that the AUC of the nomogram model was 0.872, and the AUC values of the four independent risk factors, namely hypertension, AR, OSI, and WSS were 0.579, 0.772, 0.706, and 0.769, respectively. Simultaneously, Figure 4A illustrates that the overall net benefit of the nomogram model is significantly higher than other independent predictors by the DCA method; Figure 4B illustrates that the nomogram model demonstrated good performance over the entire range of threshold by CIC analysis. The Hosmer–Lemeshow test *p*-value of 0.856 indicated a proper fit for the model. The calibration curve of the nomogram was drawn internally by a bootstrap sampling of 1,000 iterations to test the internal calibration. Figure 5A illustrates the appropriate calibration of the model. These findings suggest that the predicted probabilities of aneurysm rupture risk by the model are consistent with the actual rupture risk.

To further confirm the efficacy and applicability of the nomogram, 96 consecutive PcomA aneurysms admitted to another medical center were retrospectively analyzed for external validation. The inclusion and exclusion criteria of patients were the same as those of the training cohort. The external validation cohort yielded an AUC value of 0.867 (Figure 3B). The Hosmer–Lemeshow test *p*-value of 0.238 indicated a proper fit for the model. The calibration curve in the external validation cohort also showed a good calibration for rupture risk prediction (Figure 5B).

### A web-based dynamic nomogram

A web-based dynamic application based on the nomogram was developed (https://wei-heng.shinyapps.io/dynnomapp/). Through this application, the rupture risk of PcomA aneurysms can be calculated precisely and the results can be got quickly through the web page.

## Discussion

This research was an effort to generate a web-based dynamic nomogram to forecast the rupture risk of PcomA aneurysms. In this study, we retrospectively collected the clinical, morphological, and hemodynamic characteristics of patients with PcomA aneurysms. The four optimal factors, including hypertension, AR, OSI, and WSS, were selected by applying LASSO analysis and identified by multivariable logistic regression analysis to develop the nomogram model. In addition to internal validation, consecutive admission data were collected from another center for external validation. The results demonstrated that the nomogram model features excellent discrimination and calibration.

Most previous studies on the rupture risk of aneurysms involve multiple locations and do not focus on a single location. Research has indicated that different locations of aneurysms have different rupture risks, with the rupture risk of anterior communicating artery aneurysms being the highest and the rupture risk of middle cerebral aneurysms and internal carotid aneurysms being lower than anterior communicating artery aneurysms (22, 23). This illustrates that aneurysms at different locations may have different anatomical geometries, natural courses, and hemodynamics. Studies focusing on a single location may be more reasonable for rupture risk assessment. In this study, we established a nomogram only by collecting data from the PcomA segment. Both internal validation and external validation of other central data resulted in high AUC values, proving that the establishment of a prediction model for aneurysms at a single site may be a method to reduce bias.

The management of UIAs remains a controversial topic (24). PcomA aneurysms are most the common type of IAs and have a high incidence of rupture (25). Aneurysm clipping or endovascular treatment has certain procedural risks and complication rates Therefore, accurate prediction of the rupture risk of unruptured PcomA aneurysms is essential for aneurysm management. In a previous study, Justiina et al. found that irregular aneurysm shape, a larger aneurysm neck diameter, and AR were strongly associated with the rupture status of PComA aneurysms (26). Jiang et al. found that larger size, higher AR, BNF, and bleb formation may be related to rupture of PcomA aneurysms. Although these pieces of literature have identified independent risk factors for PcomA aneurysms rupture, there is still obvious uncertainty when these factors were used to predict aneurysm rupture alone. Aneurysm rupture is a complex process affected by multiple factors, and the nomogram can be used for multi-parameter diagnosis or prediction of disease onset or progression. Compared with single independent predictors, a nomogram can predict the risk of aneurysm rupture risk more accurately.

The PHASES system, released in 2014, recruited the largest representative cohort to predict the 5-year rupture risk of IAs. Its ease of use and high level of evidence have contributed to its popularity as a scoring system in daily clinical practice (27). However, recent studies have shown that PHASES is not accurate in predicting the risk of aneurysm rupture (28, 29). There may be some important factors that are not included in the scoring system. Hemodynamics is as important as morphology in discriminating the risk of aneurysm rupture. A correlation between hemodynamics and aneurysm rupture has been widely demonstrated in previous studies (30, 31). Therefore, it is necessary to add hemodynamic parameters in the nomogram to predict the rupture risk of IAs.

Prediction models are increasingly used to complement clinical reasoning and decision making in modern medicine. The popular method is to randomly divide the training data into two parts: one is used to develop the model and the other is used to measure its performance. A more sophisticated approach is to use cross-validation. To improve the stability of cross-validation, the whole process can be repeated many times and new sub-samples can be randomly selected. Studies have shown that the most effective validation is achieved through computer-intensive resampling techniques such as bootstrap (32, 33). Bootstrap is a resampling technique in statistics, which is used to estimate standard error, confidence interval, and deviation. The core of bootstrap is to generate a series of bootstrap pseudo-samples, where each sample is the initial data and is then put back to sampling. Through the calculation of pseudo samples, the distribution of statistics is obtained. It is proved that when the initial sample is large enough, bootstrap sampling can approach the population distribution unbiased (34).

Different from traditional multivariate logistic regression analysis, this study applied the LASSO analysis method to identify variables for reducing multicollinearity between variables, minimizing coefficients, and reducing variance. LASSO is an improvement of the multivariate logic regression analysis algorithm, which uses regularization to make the selection of variables more stringent, and finally, the model built by the selected variables is more difficult to overfit. At the same time, LASSO also brings the risk of underfitting, so this study selects variables mainly by LASSO and supplemented by multiple logical regression analysis. As a result, an optimal λ of 0.058 was adopted. Four factors with non-zero coefficients were ultimately selected by the 10-fold cross-validation to prevent overfitting, and a nomogram model was developed accordingly. The AUC in the training cohort and external validation cohort proved the appropriate discriminative ability of the nomogram. In addition, DCA showed superior overall net benefit, and CIC demonstrated good performance in the entire range of threshold probabilities. At the same time, the calibration of the nomogram was assessed through the training cohort and external validation cohort. The Hosmer–Lemeshow test was used to evaluate the accuracy of the predicted rupture risk to the actual rupture risk. The results show that the *p*-value is significant >0.05. The calibration curve also shows that the model was calibrated appropriately.

The selection of appropriate boundary conditions is crucial for the aneurysm model. The inlet is usually set to a time-dependent speed or flow waveform. In general, these values are collected by Doppler ultrasound. But in many cases, it is difficult to collect the flow characteristics of each patient. Therefore, flow rates are mostly taken from the literature (35, 36). Since the pulsatile effects are clearly smaller in the cerebrovascular compared to cardiac arteries, the impact of the profile type is negligible. If only cycle-averaged flow fields are desired (e.g., mean aneurysmal velocities), time-saving and steady state simulations may be sufficient. However, hemodynamic parameters such as OSI require time-dependent computations (37). Using flowrate-independent parameters to build predictive models is also an approach. Previous studies have shown that flowrate-independent parameters WSS and energy loss are superior to the traditional hemodynamic parameters in predicting aneurysm stability after verification by machine-learning algorithm (38).

Oscillatory shear index indicates the magnitude of WSS fluctuations and describes the tangential force oscillation as a function of the cardiac cycle. Previous studies reported that a higher OSI had significant associations with the rupture of IAs (39) or high OSI correlated to the rupture point. WSS was also a hemodynamic parameter widely studied in IAs. This study showed that low WSS was correlated with the rupture risk of PcomA aneurysms, consistent with previous studies of aneurysms at other sites (31). The mechanism of aneurysm rupture caused by low WSS or high OSI may activate inflammatory cell-mediated destructive remodeling (40). Low WSS and high OSI could upregulate endothelial surface adhesion molecules, leading to flow-induced nitrous oxide dysfunction and increasing endothelial permeability, thereby promoting atherogenesis and inflammatory cell infiltration. Enhanced endothelial permeability promotes leukocyte transmigration, and these inflammatory infiltrates abundantly produce matrix metalloproteinases to degrade the extracellular matrix, leading to the disruption of vascular integrity and thus driving intracranial aneurysm growth and rupture (41–43). Our results also suggest that a history of hypertension and higher AR increase the rupture risk of PcomA aneurysms. Previous studies suggested that hypertension may be involved in aneurysm rupture through the renin–angiotensin–aldosterone system, and the latest multicenter study demonstrated that the risk of aneurysm rupture due to hypertension could be significantly reduced using renin–angiotensin–aldosterone system inhibitors (44). A large cohort study also showed recently that higher AR was an independent predictor of aneurysm rupture (45).

The clinical data, morphological characteristics, and hemodynamic parameters of PcomA aneurysms were collected, the factors that best predict the risk of PcomA aneurysm rupture were selected through Lasso, and the nomogram was established. A nomogram model was established with favorable discrimination and calibration after internal validation and independent external validation. Then a web-based dynamic nomogram application was developed, and the medical staff could conveniently access the website through mobile phones or computers to obtain the prediction results. The purpose of this study is to supplement a practical model based on the existing models and provide additional tools for PcomA aneurysm management.

There were also several limitations in this study. Firstly, this is a retrospective study, and previous studies have shown that morphological changes may occur before and after aneurysm rupture. Therefore, prospective studies with dynamic follow-up of aneurysms may further reduce the deviation. However, in reality, the severe consequences of aneurysm rupture, the development of treatment techniques and the positive attitude of patients, and long-term dynamic follow-up is difficult to achieve. Secondly, although our nomogram model included clinical, morphological, and hemodynamic factors, there still may be other important parameters that need to be considered, such as radiomics and vessel wall enhancement, which have been found to be related to rupture risk in recent studies. Thirdly, although we performed external validation, a larger set of data would better reflect the calibration. Finally, only Chinese people were included in this study, and caution is needed when applying our results to other countries and ethnicities.

## Conclusion

The rupture risk of PcomA aneurysms was strongly associated with hypertension, high AR, high OSI, and low WSS. A web-based dynamic nomogram model was established based on LASSO's results. The nomogram model indicated excellent discrimination and calibration through internal and external validation. This nomogram can be applied for aneurysm rupture risk stratification and therapy optimization due to its effectiveness and ease of use.

## Data availability statement

The original contributions presented in the study are included in the article/supplementary material, further inquiries can be directed to the corresponding author/s.

## Ethics statement

The studies involving human participants were reviewed and approved by Clinical Research Ethics Committee of Renmin Hospital of Wuhan University and Ethics Committee of Jingzhou Central Hospital. The patients/participants provided their written informed consent to participate in this study.

## Author contributions

HW, QT, and ML contributed to the conception and design and drafted the manuscript. HW, KY, JW, PH, YG, and WH contributed to data acquisition and data analysis. HW, QT, QC, WH, and ML made the article preparation, editing, and review. All authors contributed to the article and approved the submitted version.

## Funding

This research was funded by National Natural Science Foundation of China (Grant Nos: 81971870 and 82172173).

## Conflict of interest

The authors declare that the research was conducted in the absence of any commercial or financial relationships that could be construed as a potential conflict of interest.

## Publisher's note

All claims expressed in this article are solely those of the authors and do not necessarily represent those of their affiliated organizations, or those of the publisher, the editors and the reviewers. Any product that may be evaluated in this article, or claim that may be made by its manufacturer, is not guaranteed or endorsed by the publisher.
